# Merestinib (LY2801653) inhibits neurotrophic receptor kinase (NTRK) and suppresses growth of NTRK fusion bearing tumors

**DOI:** 10.18632/oncotarget.24488

**Published:** 2018-02-13

**Authors:** Bruce W. Konicek, Andrew R. Capen, Kelly M. Credille, Philip J. Ebert, Beverly L. Falcon, Gary L. Heady, Bharvin K.R. Patel, Victoria L. Peek, Jennifer R. Stephens, Julie A. Stewart, Stephanie L. Stout, David E. Timm, Suzane L. Um, Melinda D. Willard, Isabella H. Wulur, Yi Zeng, Yong Wang, Richard A. Walgren, Sau-Chi Betty Yan

**Affiliations:** ^1^ Lilly Research Laboratories, Eli Lilly and Company, Indianapolis, IN 46285, USA

**Keywords:** NTRK fusion, merestinib, LY2801653, NTRK inhibitor, type II kinase inhibitor

## Abstract

Merestinib is an oral multi-kinase inhibitor targeting a limited number of oncokinases including MET, AXL, RON and MKNK1/2. Here, we report that merestinib inhibits neurotrophic receptor tyrosine kinases NTRK1/2/3 which are oncogenic drivers in tumors bearing NTRK fusion resulting from chromosomal rearrangements. Merestinib is shown to be a type II NTRK1 kinase inhibitor as determined by x-ray crystallography. In KM-12 cells harboring *TPM3-NTRK1* fusion, merestinib exhibits potent p-NTRK1 inhibition *in vitro* by western blot and elicits an anti-proliferative response in two- and three-dimensional growth. Merestinib treatment demonstrated profound tumor growth inhibition in *in vivo* cancer models harboring either a *TPM3-NTRK1* or an *ETV6-NTRK3* gene fusion. To recapitulate resistance observed from type I NTRK kinase inhibitors entrectinib and larotrectinib, we generated NIH-3T3 cells exogenously expressing *TPM3-NTRK1* wild-type, or acquired mutations G595R and G667C *in vitro* and *in vivo*. Merestinib blocks tumor growth of both wild-type and mutant G667C *TPM3-NTRK1* expressing NIH-3T3 cell-derived tumors. These preclinical data support the clinical evaluation of merestinib, a type II NTRK kinase inhibitor (NCT02920996), both in treatment naïve patients and in patients progressed on type I NTRK kinase inhibitors with acquired secondary G667C mutation in NTRK fusion bearing tumors.

## INTRODUCTION

Aberrant genomic rearrangements frequently lead to uncontrolled oncogenic driven growth and are generally insensitive to standard anti-cancer modalities. Although occurrence of genomic rearrangements in solid tumors are rare overall, specific cancers such as prostate and Ewing Sarcoma show prevalence of gene fusions in 50-70% and 90% of cases, respectively [1]. Therefore, pharmacologically targeting gene fusions is of great interest for drug development. Targeted treatments for some of the gene fusions involving the kinase domain of ALK, ROS1, and ABL have been successful and approved [2–4].

The neurotrophic receptor tyrosine kinases 1, 2 and 3 (NTRK1, 2, 3 also known as TrkA, B, C) are chiefly involved in neuronal development [5]. Yet, genomic rearrangements involving the NTRK kinase domain fused to unrelated 5’ gene partners causing oncogenic tumor growth are evident in many adult and pediatric cancers with various frequencies. Up to 3% of NSCLC patients harbor an *NTRK1* fusion while 90% incidence of an *NTRK3* fusion are reported in rare tumors such as congenital fibrosarcoma and mammary analogue secretory carcinoma (MASC) [6]. NTRK fusions as oncogenic drivers are further evidenced by clinical treatment response case series to compounds with pan-NTRK activity such as crizotinib, entrectinib (RXDX-101) and larotrectinib (LOXO-101) [7–11].

Merestinib (LY2801653) is an orally bioavailable, small molecule kinase inhibitor targeting several oncokinases, currently in phase II clinical development (NCT02711553). Previously reported as a type II MET kinase inhibitor, merestinib is also potent against several oncokinases such as MST1R (aka RON), AXL, MERTK, MKNK1/2, and ROS1 [12]. We report here that merestinib is also a type II NTRK1 kinase inhibitor as determined by x-ray crystallography. We further report that merestinib inhibits NTRK1, 2, 3 *in vitro* and *in vivo* models expressing *NTRK1* or *NTRK3* rearrangements. Acquired resistance to entrectinib or larotrectinib treatment with secondary mutation at G667C or G595R in NTRK1 kinase domain has been reported [8–10]. As a type II NTRK1 kinase inhibitor, merestinib is shown in this study to retain potency *in vitro* and *in vivo* in NIH-3T3 cells engineered to carry *TPM3-NTRK1* with a kinase domain G667C mutation.

## RESULTS

### Biochemical assessment of merestinib and its metabolites

**Figure 1:**
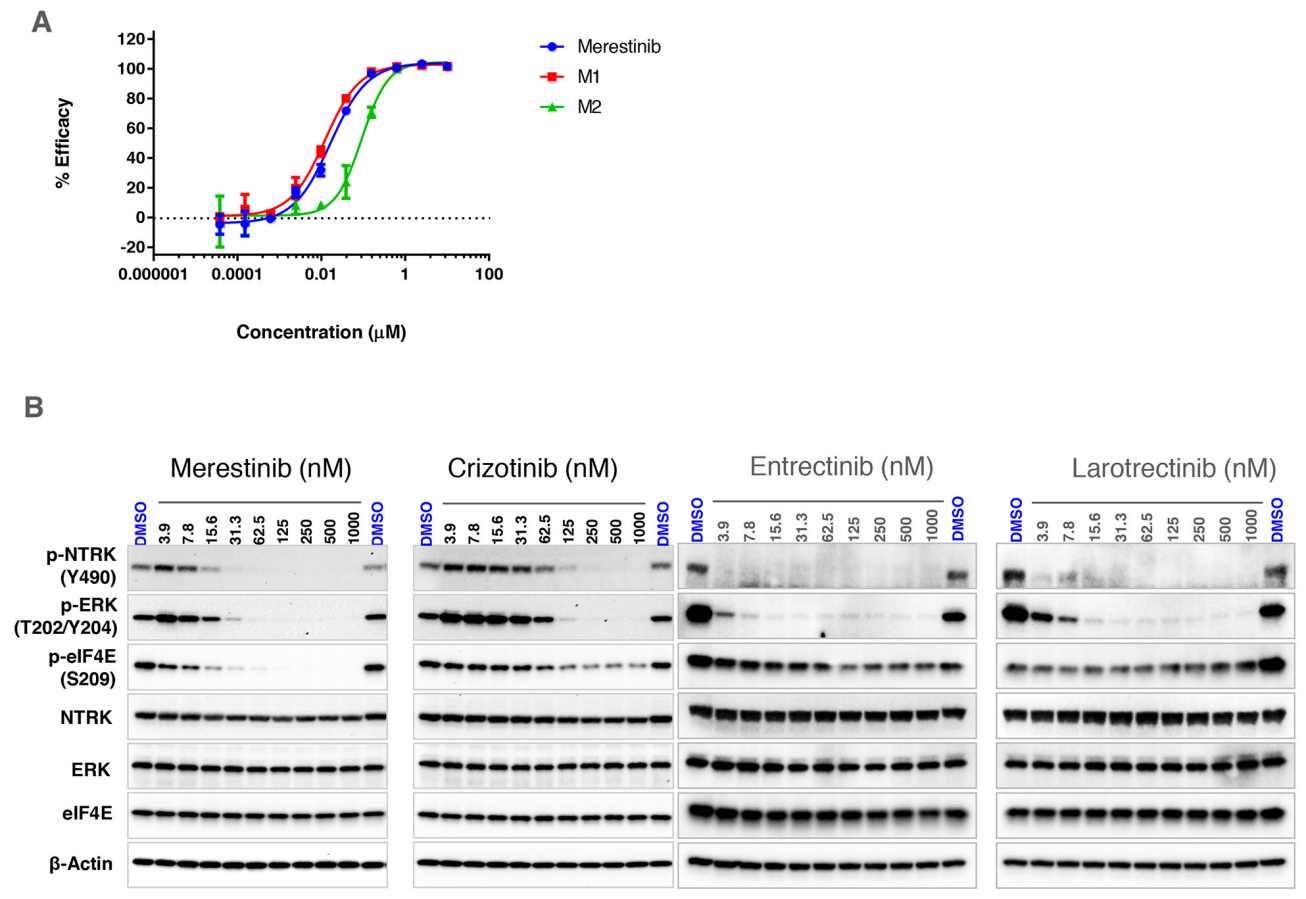
*In vitro* effect of merestinib treatment on cell-based NTRK1 inhibition **(A)** Effect of merestinib and its two metabolites (M1, M2) on cell-based PathHunter TrkA inhibition. Ten-point IC_50_ analysis of inhibitor ranging from 3.8 nM - 10 μM was performed. **(B)** Western blots of KM-12 cells treated with merestinib, crizotinib, entrectinib and larotrectinib *in vitro*. Cells were treated with compound ranging from 3.9 - 1000 nM for 2 hours. Western blots were re-probed for total NTRK, ERK, eIF4E and β-actin for loading control purposes.

Merestinib was previously identified to inhibit MET kinase biochemically at an IC_50_ of 4.7 nM with cell based activity IC_50_ values ranging between 35-52 nM. Additional kinase targets of merestinib including AXL, MERTK, TYRO3, ROS1 and MKNK1/2 were also inhibited in cell-based assays ranging between 0.1-170 nM [12]. Two primary metabolites of merestinib were observed in a phase I clinical study (NCT02779738), designated M1 and M2 (structures shown in Supplementary Figure 1). To address whether M1 and M2 exhibited similar kinase profile activity as merestinib, both metabolites were tested at concentrations of 0.2, 1 and 5 μM using 468 kinase panel scanMAX. Indeed, both metabolites showed similar inhibitory activity to that of merestinib (Supplementary Table 1). Merestinib and both metabolites inhibited NTRK1, 2, 3. Binding constants (Kd) calculated for NTRK1 for merestinib, M1 and M2 were 20, 15, 120 nM, respectively; NTRK2 binding constants were 92, 61, 320 nM, respectively and for NTRK3 were 54, 41, and 160 nM, respectively. *In vitro* inhibition of cell-based NTRK1 analysis (activation by the ligand NGF - PathHunter^®^) showed an IC_50_ for merestinib, M1 and M2 at 17, 12, 92 nM, respectively (Figure 1A).

### *In vitro* effects of merestinib and its metabolites in KM-12 cells

The colorectal carcinoma cell line KM-12 created in 1988 [13], was later discovered as having an intrachromosomal translocation in chromosome 1 [14] fusing the actin-binding protein, tropomyosin, to the NTRK kinase domain forming a constitutively active *TPM3-NTRK1* gene fusion. The coil-coil domain of the TPM3 is hypothesized to induce dimerization of the NTRK fusion protein leading to constitutive NTRK activation in the absence of its ligand NGF. To examine if merestinib inhibits NTRK1 phosphorylation *in vitro*, KM-12 cells were treated for 2 hours ranging in concentration from 3.9 -1000 nM. Merestinib showed a dose dependent decrease in p-NTRK1 Y490 resulting in complete inhibition at 62.5 nM as determined by western blot (Figure 1B). Crizotinib, which is reported to inhibit NTRK1 [15], blocked Y490 phosphorylation to near completion at 250 nM. (Figure 1B). Both merestinib and crizotinib showed dose dependent inhibition of phosphorylated MAPK 42/44 (ERK) in concordance with their respective p-NTRK downstream signaling (Figure 1B). In comparison, both entrectinib and larotrectinib totally inhibited NTRK1 Y490 phosphorylation at 3.9 nM and the phosphorylation of MAPK 42/44 (ERK) at 15.6nM (Figure 1B).

Merestinib is a potent direct inhibitor of MKNK1/2, the kinases responsible for phosphorylating eIF4E at S209 [16]. In KM-12 cells, merestinib reduced p-eIF4E levels with near-complete inhibition at 62.5 nM (Figure 1B). Crizotinib, entrectinib and larotrectinib displayed a small reduction of p-eIF4E at the higher concentrations, suggesting either these three kinase inhibitors mildly inhibit MKNK1/2 or the reduction of p-eIF4E might have resulted from downstream signaling from inhibiting p-NTRK1. Consistent with biochemical scanMAX data, merestinib, its metabolite M1, and slightly less so for metabolite M2 also displayed potent inhibition of p-NTRK1, p-ERK and MKNK1/2 (as a reduction of p-eIF4E) in KM-12 cells (Supplementary Figure 2).

We further examined if merestinib, M1 and M2 metabolites suppress KM-12 cell proliferation *in vitro*. Within 72 hours, treatment with merestinib, M1, or M2 suppressed cell proliferation with an IC_50_ of 10 nM, 16 nM and 102 nM, respectively. Crizotinib also displayed an anti-proliferative response with an IC_50_ of 92 nM (Supplementary Figure 3A). To assess anchorage independent growth, KM-12 cells embedded in alginate were treated with inhibitor for 72 hours and assessed for colony formation. Merestinib, M1 and M2 treatment decreased anchorage independent growth with an IC_50_ of 45 nM, 79 nM and 206 nM, respectively, relative to crizotinib (IC_50_ =276 nM) (Supplementary Figure 3B). Collectively, these data suggest that merestinib and the metabolites M1 and M2 block both anchorage dependent and independent cell growth in *TPM3-NTRK1* bearing KM-12 cells.

### Merestinib anti-tumor activity in two TPM3-NTRK1 harboring xenograft tumor models

**Figure 2:**
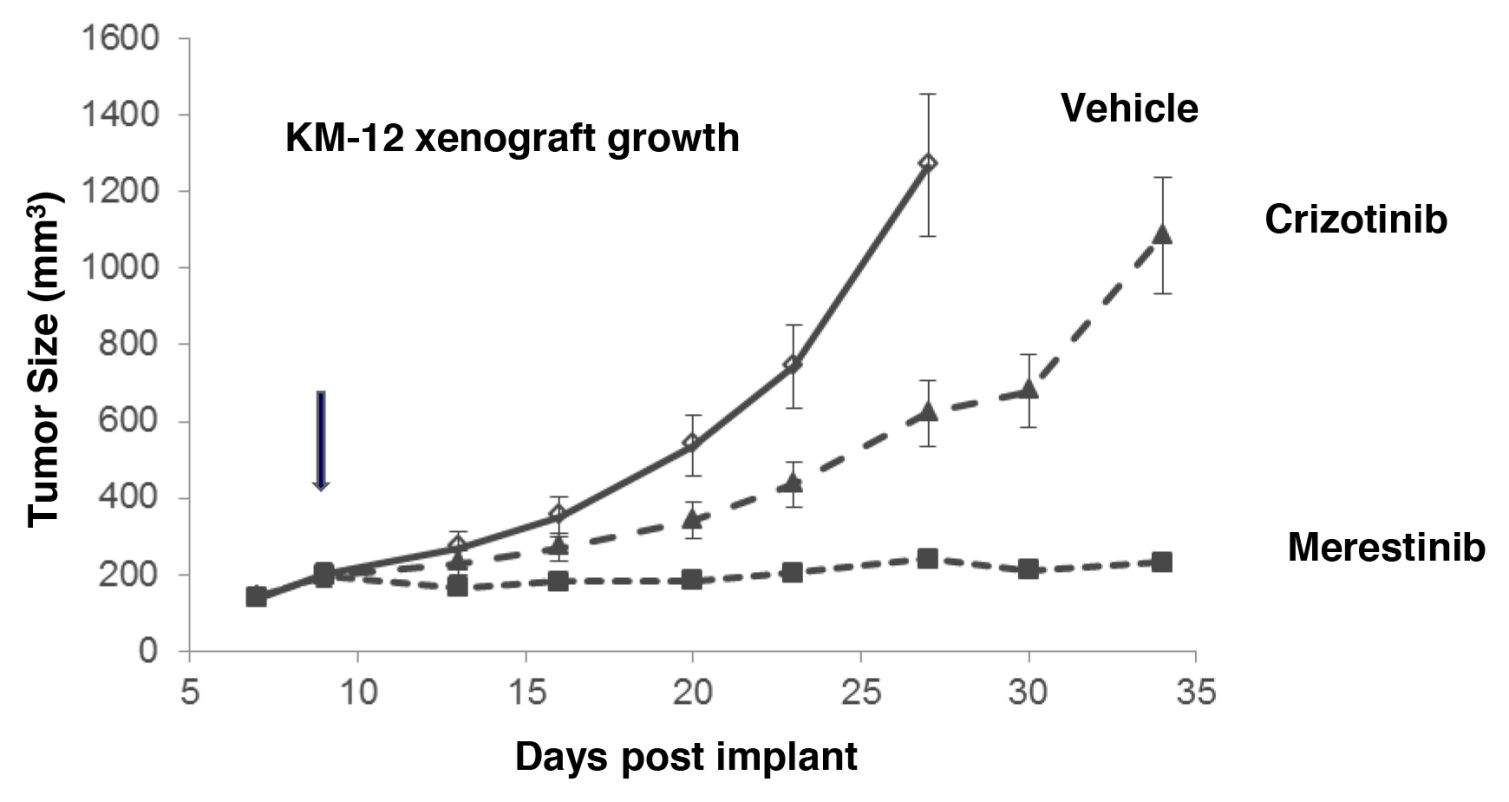
Anti-tumor activity of merestinib in KM-12-derived xenograft tumors Merestinib was dosed orally once daily at 24 mg/kg (- -■- -), crizotinib was dosed orally twice daily at 25 mg/kg (- -▲- -). Dosing began in athymic nude mice implanted with KM-12 cells once average tumor burden reached 150-200 mm^3^ on Day 9 (denoted by arrow). Vehicle (— ◊—) dosing terminated on Day 27 as animals in this group were removed due to excessive tumor burden. Statistical analyses comparing vehicle to the two treated cohorts were performed on Day 27, and comparing crizotinib and merestinib (p<0.001) on Day 34. Animal weights were measured twice weekly with no significant weight alteration relative to vehicle.

**Figure 3:**
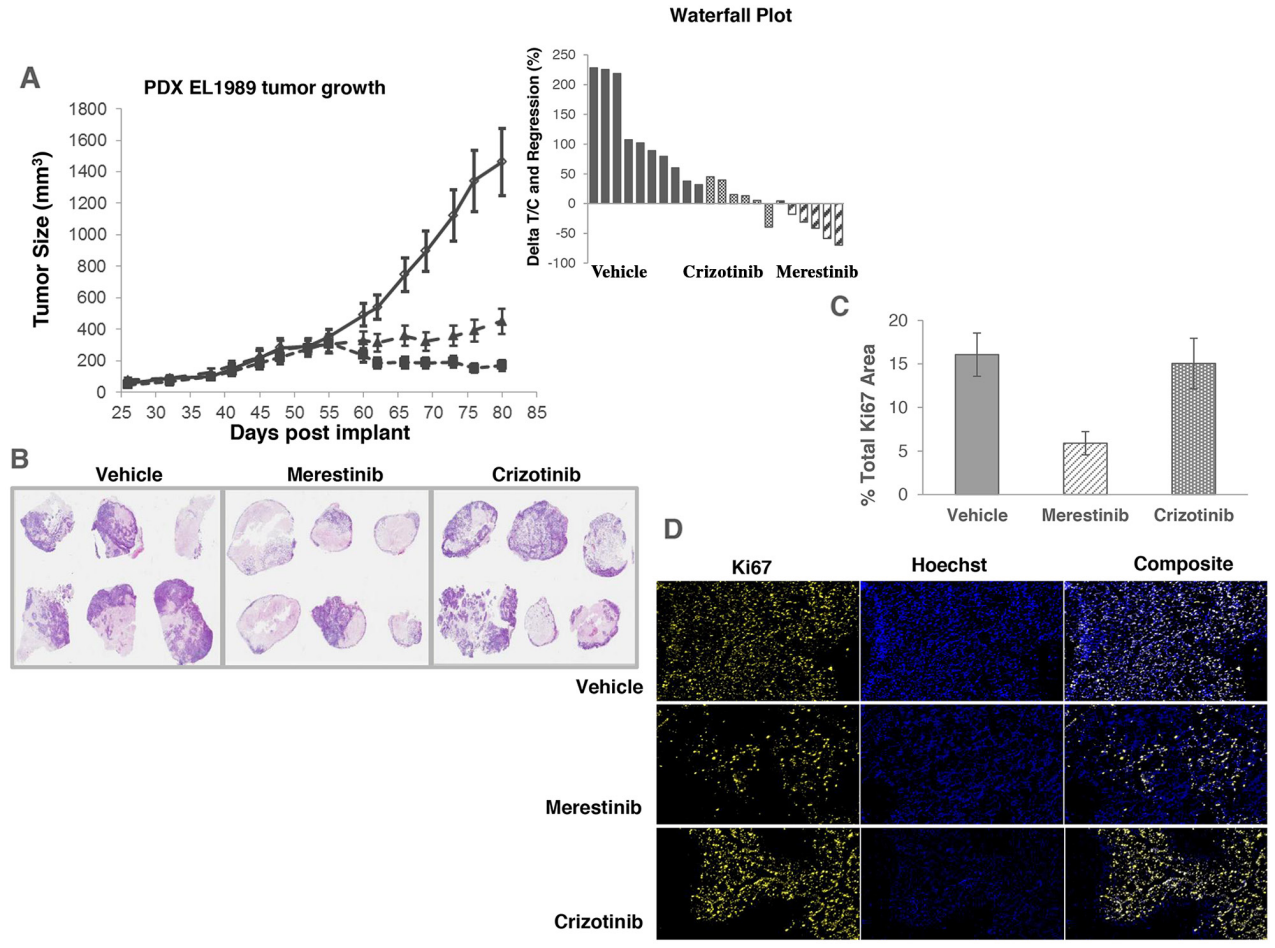
Anti-tumor activity of merestinib in patient-derived xenograft tumors (EL1989) bearing *TPM3-NTRK1* fusion **(A)** Merestinib was dosed daily at 24 mg/kg (- -■- -), and crizotinib was dosed twice daily at 25 mg/kg (- -▲- -). All treatment cohorts began dosing on Day 52. Waterfall plot depicts individual animal tumor response to treatment as measured after 28 days of dosing (on Day 80). Graph bars below the x-axis indicate tumor regression. **(B)** Low magnification image (6x) of hematoxylin and eosin stained EL1989 PDX tumor histological sections grouped by treatment and harvested at the end of the study: Vehicle control tumors, merestinib treated tumors, crizotinib treated tumors. Viable tumor tissue stains light to dark purple, areas of necrosis stain as pale pink and areas of mucin accumulation are very pale or lack staining. Tumor viability, tumor necrosis and mucin accumulation scoring were performed by a board certified pathologist (KMC). Estimated % viable tumor tissue per cohort are as follows: Vehicle, mean 50%, range 5-90%; merestinib, mean 25%, range 5-70%; crizotinib, mean 50%, range 5-90%. Refer to Supplementary Table 2 for individual assessments. **(C)** Percent proliferating tumor cells by treatment based on Ki67 immunostaining and image analysis. Merestinib treated tumors (n=6) were significantly reduced (p=0.016) relative to vehicle (n=10). Crizotinib treated tumors were not significantly different (p=0.94). Five of six merestinib treated tumors showed thin rims of viable tumor tissue located mostly around the outer perimeter in comparison to the crizotinib treated cohort that displayed tumors with more abundant viable cells distributed throughout most tumor sections. Histological sections from vehicle control tumors were performed only on portions of the whole tumors rather than whole intact samples. Of these sections, viable cells were distributed throughout the tumors similar to crizotinib treated tumors. Tissue sections were stained with Ki67, and imaged and analyzed using an iCys laser scanning cytometer. Error bars denote SEM. **(D)** Representative image of the Ki67 immunostaining of the tumors from vehicle control, crizotinib and merestinib treated groups.

As previously reported that *TPM3-NTRK1* drives tumor growth in KM-12 cells [14], we sought to assess merestinib activity *in vivo* in KM-12 xenograft tumors in athymic nude mice. Merestinib (24 mg/kg once daily orally) or crizotinib treatment (25 mg/kg twice daily orally) resulted in significant anti-tumor effect (T/C=4%, p<0.001; T/C=39.5%, p<0.001 respectively) as compared with vehicle control (Figure 2), with the anti-tumor activity of merestinib statistically different than that of crizotinib (p<0.001). These data suggest a correlation between p-NTRK1 reduction and reduced tumor burden. To investigate further, anti-tumor effect of merestinib was also evaluated in a patient tumor-derived xenograft (PDX) model EL1989. EL1989 was from a colorectal carcinoma and was characterized to harbor the same *TPM3-NTRK1* gene rearrangement as the KM-12 cell line (Supplementary Figure 4). Merestinib dosed daily orally at 24 mg/kg led to tumor regression within 8 days of treatment initiation (-15.4%, p=0.003) and persisted to end of treatment (-39.1%, p<0.001). Crizotinib dosed twice daily orally at 25 mg/kg achieved tumor stasis, but without tumor regression (T/C =13.5%, p<0.001) (Figure 3A). Merestinib treatment showed more anti-tumor effect than crizotinib treatment (Day 80, p =0.001). Together, these data indicate that merestinib shows compelling *in vivo* anti-tumor effect in *TPM3-NTRK1* bearing colorectal carcinoma tumors.

EL1989 tumor was found to have mucinous secretion (Figure 3B) as illustrated in the H&E staining of the vehicle group tumors showing accumulation of pale foamy cytoplasmic vacuoles in tumor cells and minimal to abundant accumulation of mucin extracellularly. Thus, H&E staining of histological sections of tumors from each cohort harvested at the end of treatment were semi-quantitatively scored for tumor cell viability, necrosis and mucin content (Figure 3B, Supplementary Table 2). In the merestinib treated cohort, mean tumor cell viability was 25% (range 5-70%) relative to either the vehicle or crizotinib cohorts, both with a mean cell viability of 50% (range 5-90%). Merestinib treatment resulted in diminished cell proliferation (measured by Ki67 immunostaining) by 63%, (p=0.017) (Figure 3C, 3D) relative to vehicle control while crizotinib was virtually unchanged. In the merestinib treated cohort, some viable adenocarcinoma tissue remained; however, significant mucin accumulation was readily apparent thus contributing to the overall residual volume, explaining why the observed tumor regression plateaued at approximately 200 mm^3^.

**Figure 4:**
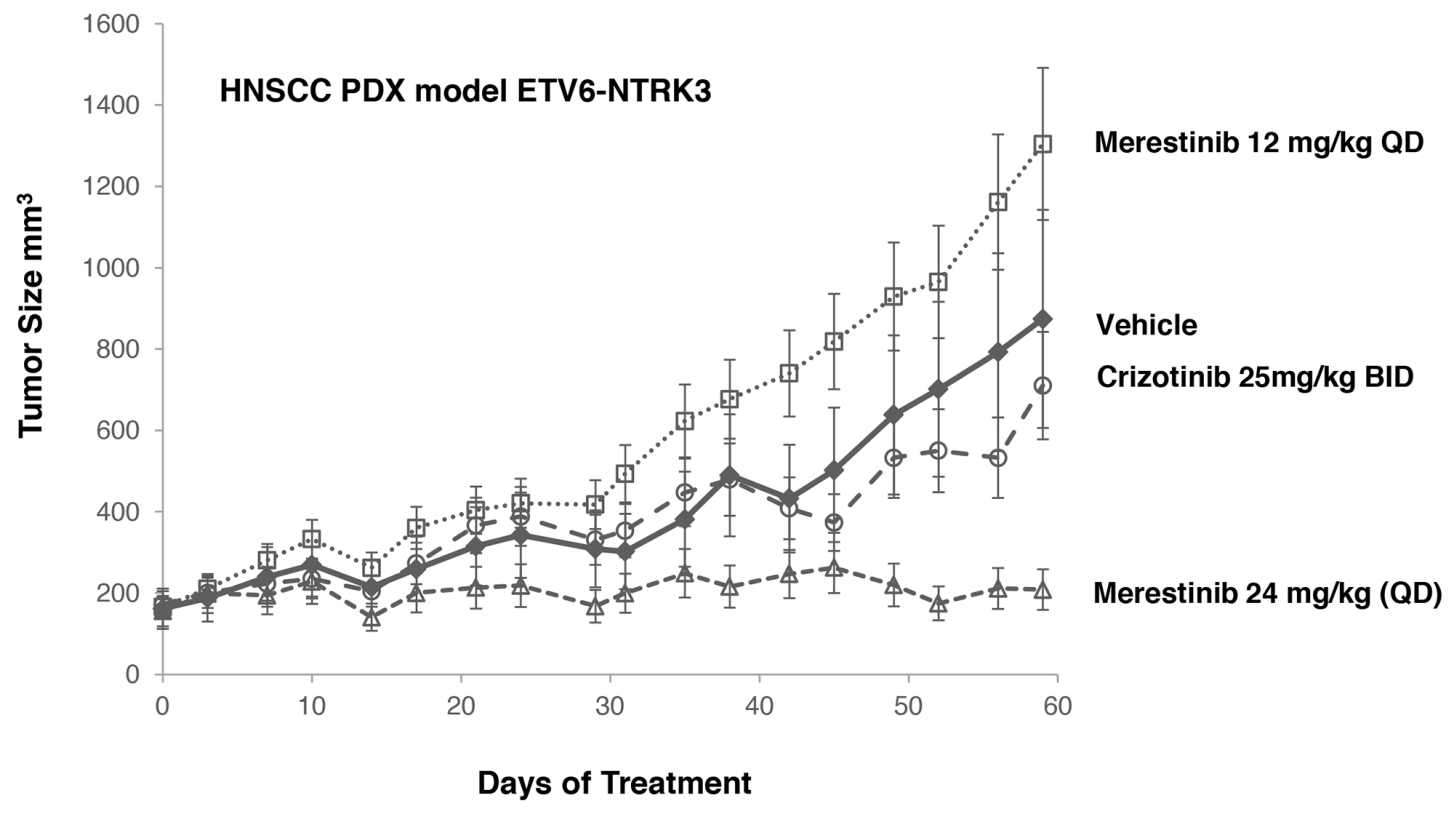
Anti-tumor effect of merestinib or crizotinib in HNSCC PDX model HNSCC PDX model harboring an *ETV6-NTRK3* fusion treated with merestinib or crizotinib (n=5 per group). Treatment began on Day 0. Merestinib dosed at 24 mg/kg once daily blocked tumor growth of the PDX HNSCC model harboring an *ETV6-NTRK3* fusion on Day 59 (T/C = 6.2%, p<0.001). Crizotinib dosed twice daily at 25 mg/kg was not statistically different than vehicle control (T/C=76.9%, p=0.525). Merestinib dosed 24 mg/kg was significantly more efficacious than crizotinib (p<0.001). 12 mg/kg merestinib failed to suppress tumor growth. Animal weights were measured twice weekly with no statistical change relative to vehicle control.

A leukemia cell line MO-91 harboring *ETV6-NTRK3* gene fusion was used by others to evaluate NTRK inhibitors [9, 15]. Crizotinib was found to inhibit the proliferation of MO-91 cells *in vitro* with an IC50 of 10 nM [15] and induced tumor regression of the MO-91 xenograft tumors at 50 mg/kg once daily dosing [15]. As MO-91 cells are not available in publicly accessible cell banks, and the parental MO-91 cells are poorly tumorigenic [15], we elected to evaluate merestinib and crizotinib in a HNSCC PDX model also bearing *ETV6-NTRK3* gene fusion (Figure 4, Supplementary Figure 5). Merestinib dosed daily at 24 mg/kg showed reduction of tumor growth relative to vehicle control (T/C=6.2%, p < 0.001) while crizotinib dosed 25 mg/kg twice daily did not show significant tumor growth reduction (T/C=76.9%, p=0.525) relative to vehicle. Together, merestinib displays potent anti-tumor effect in tumors with NTRK fusions.

### Merestinib inhibits p-NTRK1 in entrectinib-resistant models *in vitro*

**Figure 5:**
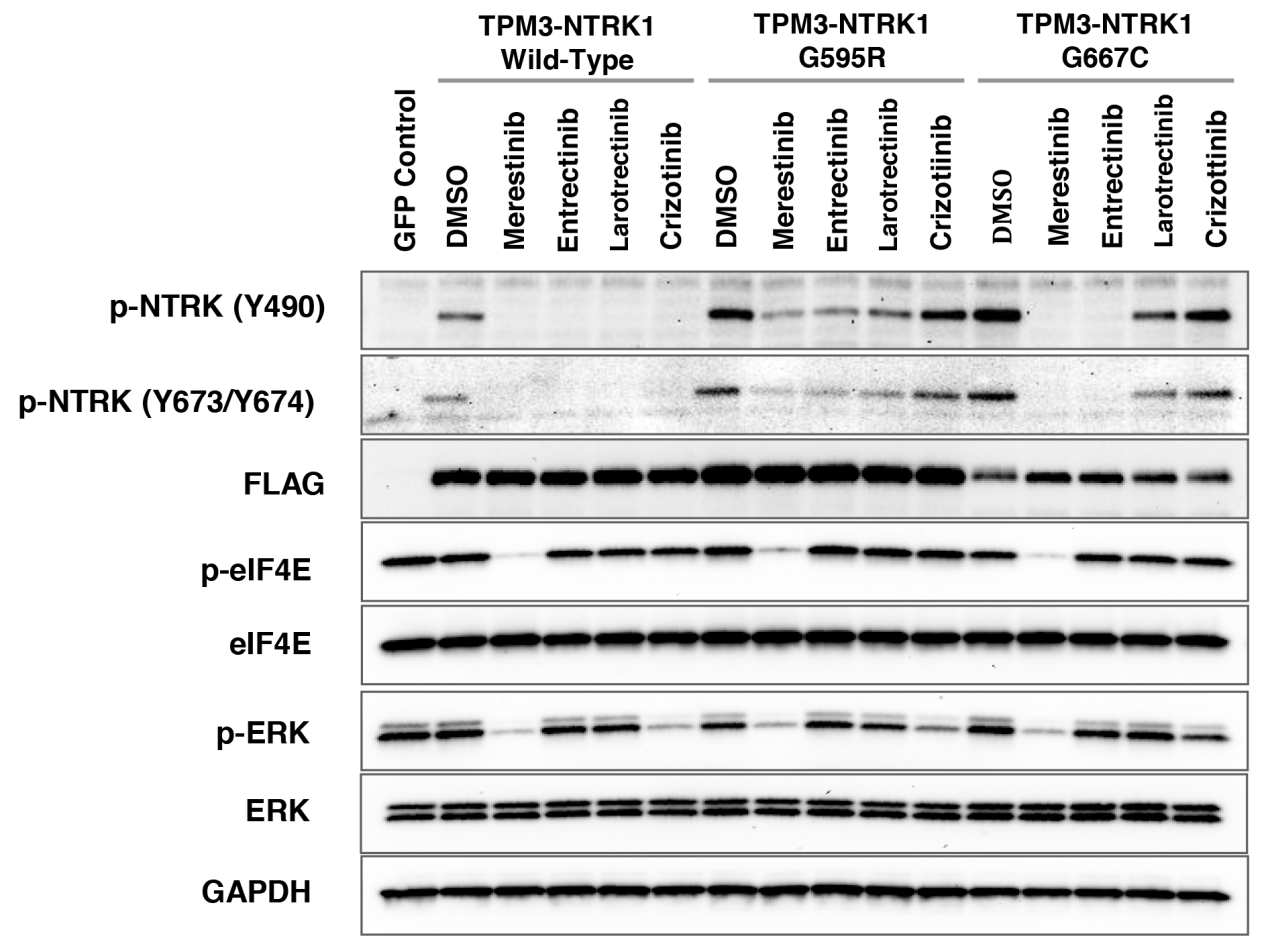
Evaluation of NTRK inhibitors with NIH-3T3 cells transfected with G595R or G667C mutation in *TPM3-NTRK1* fusion *in vitro* Cell lysates from NIH-3T3 cells stably transfected with *TPM3-NTRK1* wild-type, mutant G595R, or G667C *TPM3-NTRK1* expressing clones were analyzed by immunoblotting after treatment with 0.5 μM of the indicated NTRK inhibitor for 4 hours. All three *TPM3-NTRK1* clones expressed 3’-3X-FLAG-Tag as confirmed by anti-FLAG antibody. eGFP control vector served as a control with no NTRK or FLAG expression.

Inhibitors targeting NTRK-gene fusions such as entrectinib [17] and larotrectinib (LOXO-101) [6] are currently in clinical trials in cancer patients harboring NTRK fusions. Acquired resistance to entrectinib or larotrectinib treatment in patients has been reported [8–10]. Two missense mutations located within the kinase domain of NTRK1, G595R and G667C were identified. These acquired secondary mutations may create steric hindrance to the binding of these two compounds, thus diminishing their potency in inhibiting p-NTRK1 activity [10]. In order to determine if merestinib reduces NTRK1 phosphorylation in either G595R or G667C *TPM3-NTRK1* mutants, stably expressing *TPM3-NTRK1* in NIH-3T3 cells with wild-type NTRK1 kinase, G595R or G667C missense mutations were created (Supplementary Figure 6). All three constructs included a 3’-terminal 3X-FLAG Tag. As expected, p-NTRK1 (Y490) signaling was reduced in the NIH-3T3 cells expressing wild-type *TPM3-NTRK1*, after treatment with 0.2 μM and 0.5 μM merestinib, entrectinib, larotrectinib or crizotinib (Supplementary Figure 7, Figure 5). NIH-3T3 cells expressing mutant G595R *TPM3-NTRK1* showed diminished p-NTRK1 upon merestinib or entrectinib treatment, but it was not completely abolished. Larotrectinib also showed partial p-NTRK1 inhibition while crizotinib had little effect. However, the NIH-3T3 G667C *TPM3-NTRK1* mutant clone remained sensitive to merestinib and entrectinib treatment, but not to larotrectinib or crizotinib. Of note, entrectinib eliminated p-NTRK1 at both Y490 and Y674/5 in the G667C mutant expressing cells in this study, whereas Russo and colleagues [10] showed very little p-NTRK1 inhibition in the G667C mutant with entrectinib as determined by Y674/Y675 phosphorylation.

In the NIH-3T3 cells with wild-type *TPM3-NTRK1* or G667C *TPM3-NTRK1*, merestinib showed almost total reduction in p-eIF4E (Figure 5), similar to that in the KM-12 cells (Figure 1). Crizotinib, entrectinib and larotrectinib did not show reduction in p-eIF4E (Figure 5). Among the four NTRK inhibitors evaluated, merestinib showed the most reduction in p-ERK in the NIH-3T3 cells with wild-type *TPM3-NTRK1*, G595R *TPM3-NTRK1* and G667C *TPM3-NTRK1* (Figure 5).

### Merestinib inhibits growth of wild-type and G667C *TPM3-NTRK1* expressing tumors *in vivo*

**Figure 6:**
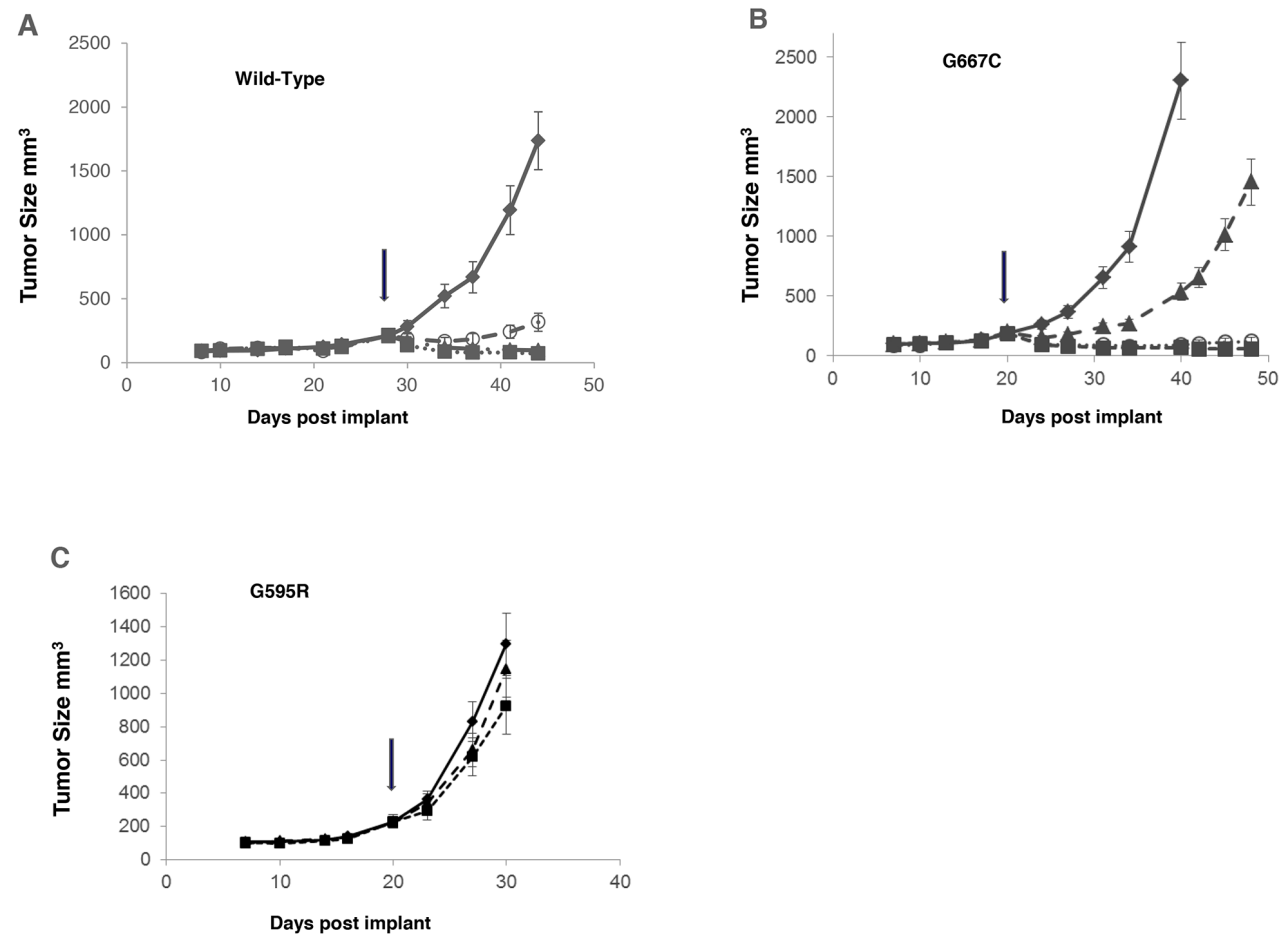
Comparison of anti-tumor effect of merestinib with entrectinib *in vivo* in tumors bearing G595R or G667C mutation NIH-3T3 cells constitutively expressing *TPM3-NTRK1* variants were implanted subcutaneously in the flank region in athymic nude mice. Once the average tumor volume reached 150-200 mm^3^, compound dosing was initiated: merestinib dosed orally once daily at 12 mg/kg (- -○- -), 24 mg/kg (- -■- -) or entrectinib dosed orally twice daily at 30 mg/kg (- -▲- -) for 21 days. Tumor growth of vehicle control (—♦—), merestinib or entrectinib treatment was evaluated in: **(A)** wild-type *TPM3-NTRK1*; **(B)** mutant G667C *TPM3-NTRK1*; **(C)** mutant G595R *TPM3-NTRK1* in mouse tumor models. Arrows indicate beginning of dosing.

Merestinib (dosed once daily at 12 mg/kg or 24 mg/kg) and entrectinib (dosed twice daily at 30 mg/kg) were evaluated in mouse tumor models with NIH-3T3 cells constitutively expressing wild-type *TPM3-NTRK1*, *TPM3-NTRK1* with G595R or G667C mutation. Both merestinib and entrectinib treatment resulted in tumor regression in tumors expressing wild-type *TPM3-NTRK1* (Figure 6A). Similar extent of tumor regression was observed in both doses of merestinib treated cohorts in animals bearing tumors with the G667C mutant within 4 days of treatment initiation (12 mg/kg once daily, regression = 46.8%, p < 0.001; 24 mg/kg once daily, regression = 51.3%, p < 0.001) and maintained through the study period. Entrectinib at 30 mg/kg twice daily dosing, showed slight tumor regression within the first 4 days of treatment initiation (regression = 19.5%, p <0.028), but this response was transient as tumors grew out while on treatment (Figure 6B). Tumors expressing mutant G595R *TPM3-NTRK1* were insensitive to either merestinib (T/C=65.2%, p=0.147) or entrectinib (T/C=86.2%, p=0.596) treatment (Figure 6C).

### Merestinib as type II NTRK1 kinase inhibitor

**Figure 7:**
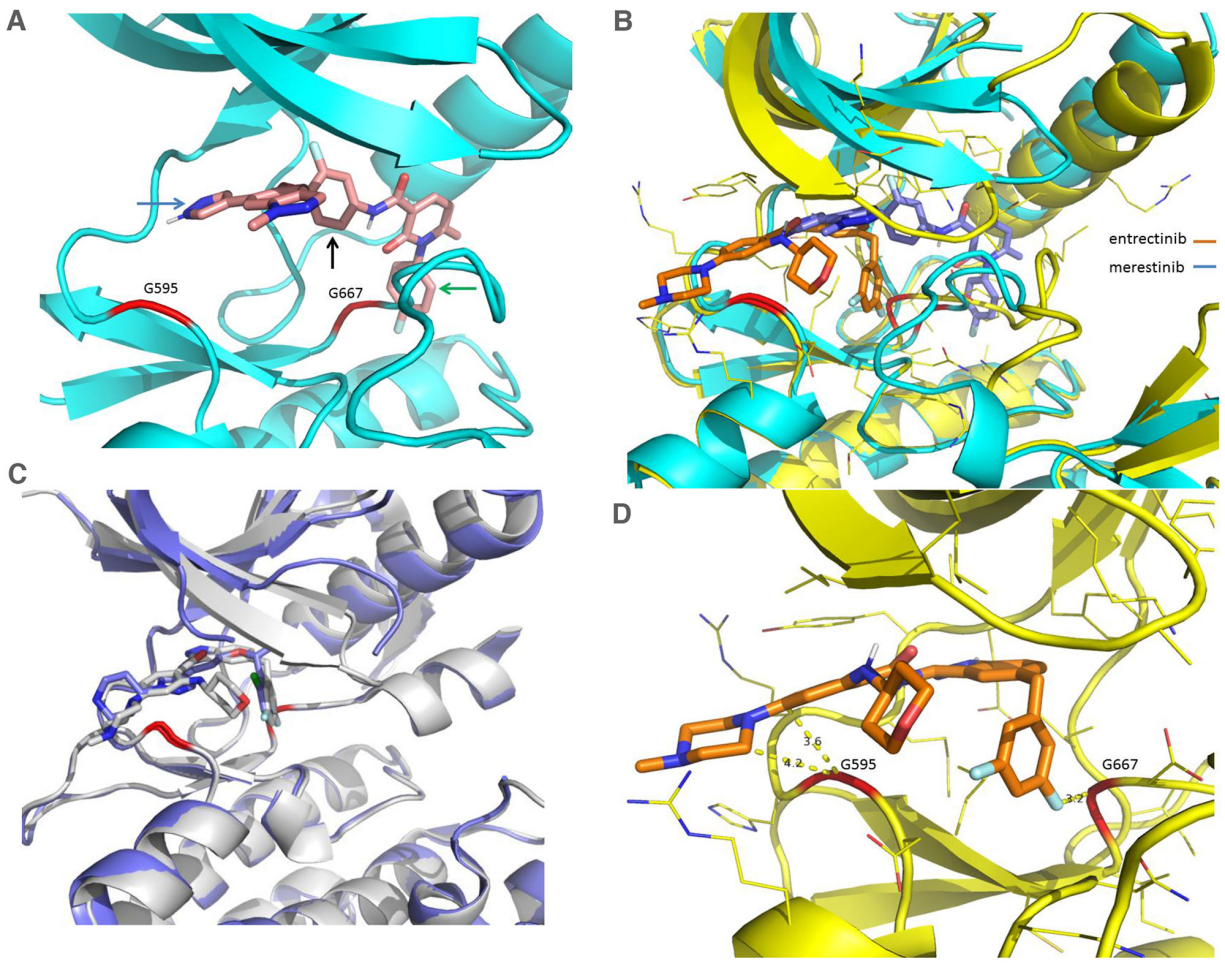
Comparison of X-Ray crystal structures of NTRK1 bound to merestinib and entrectinib, and structures of ALK bound to entrectinib and crizotinib **(A)** Merestinib bound to NTRK1 in ribbon diagram where the protein part is in cyan and the inhibitor in pink. G595 and G667 are highlighted in red in the ribbon. Arrows are used to show key interactions between the inhibitor and protein, the blue arrow for hinge interaction, the black arrow for hydrophobic interaction in the interior pocket while the green arrow for the interaction in the pocket created from the DFG-out conformation. **(B)** Entrectinib (in brown) and merestinib (in blue) bound to NTRK1 (complex with entrectinib in yellow and complex with merestinib in cyan). The dramatically different conformations in the activation loop (downstream G667) arise from the DFG-in conformation with the type I inhibitor entrectinib and the DFG-out conformation with the type II inhibitor merestinib. **(C)** Entrectinib (in grey, PDB accession code 5fto) and crizotinib (in blue, PDB accession code 2xp2) bound to ALK to show the similar binding mode of the type I inhibitors. **(D)** Entrectinib bound to NTRK1 with shown close distances to G595 and G667. The short distance between G595, G667 and the inhibitor means that mutations to a bulkier residue (G595R, G667C) will disturb the bound inhibitor. Larotrectinib is also a type I inhibitor with a similar fluoro-phenyl group located near G667C and thus is expected to be sensitive to the same mutation. This is actually what was presented in a recent study [9].

Merestinib was co-crystalized with NTRK1 kinase and was shown to bind to the DFG-out configuration of NTRK1 (Figure 7A, 7B), as was previously shown for the binding mode of merestinib to the MET kinase domain [12], confirming merestinib is a type II kinase inhibitor of NTRK1. Of note, the binding configuration of the warhead portion of merestinib in NTRK1 differs with respect to the binding configuration with MET kinase domain [12]. The location of the two acquired resistant mutations from entrectinib and larotrectinib treatment, G595 and G667 (highlighted in red) in the structure of the NTRK1 kinase domain (Figure 7A), is far from the bound merestinib with a distance of at least 5 Å between the Cα and most of the atoms from the bound merestinib. The binding conformation of merestinib (in blue) differs from that of entrectinib (in brown) in the NTRK kinase pocket (Figure 7B). The different conformations are largely the result of entrectinib being a type I inhibitor (with DFG-in conformation) and merestinib being a type II inhibitor (with DFG-out conformation). Larotrectinib is a type I inhibitor and its conformation bears more similarity to that of entrectinib as reported by Drilon et al. [9]. Crizotinib, a type 1B inhibitor of ALK [18], and entrectinib, a type I inhibitor of ROS1 [19], are expected to bind much closer in proximity to G595 and G667 of NTRK1, as seen from the structures of their complex with NTRK1 (internal data) or projected from their complex with ALK based on homology (Figure 7C). The G667 position of NTRK1 corresponds to G1269 in ALK and is frequently mutated to alanine. Due to this close proximity to this mutation site, crizotinib lost 6-8 fold potency in G1269A mutation in ALK [20].

Entrectinib is bound to NTRK1 with short distances to G595 and G667 (Figure 7D). The short distance between G595, G667 and the inhibitor means that mutations to a bulkier residue (G595R, G667C) will disturb the bound inhibitor. Larotrectinib is also a type I inhibitor with a similar fluoro-phenyl group located near G667C and thus is expected to lose sensitivity to the same mutation, as was shown in a recent study [9]. Merestinib does not have a group very close to either G595 or G667, and therefore lost activity to a lesser degree. We believe that the different sensitivity shown by merestinib towards the NTRK1 G595R and G667C mutations is largely due to its different binding conformation, especially as a type II kinase inhibitor of NTRK1. While protein conformational plasticity and inhibitor flexibility can accommodate the mutations to some degree, merestinib depends much less on such accommodation, particularly at the G667C site.

## DISCUSSION

Considerable research has been conducted in targeting the NTRK1-NGF axis in drug development for pain management [23–25] and for targeting NTRK1, 2, 3 as oncogenes in 19 different types of cancer [6, 21, 22]. In this report, merestinib is shown to be a type II NTRK1 kinase inhibitor based on the x-ray crystal structure analysis, as it binds to the DFG-out configuration of the NTRK1 kinase domain. Merestinib and its primary metabolites, M1 and M2, are potent inhibitors of the NTRK kinases with merestinib having a Kd to NTRK1, 2, 3 of 20, 92 and 54 nM, respectively. Merestinib and its metabolites inhibit p-NTRK1 (Y490) in a dose-dependent manner in the colorectal KM-12 cell line harboring a *TPM3-NTRK1* fusion. Phosphorylation at Y490/Y785 reportedly activates downstream MAPK signaling [26, 27], which supports the observed reduction of p-ERK in KM-12 cells upon treatment with merestinib or its metabolites.

Merestinib and the metabolites inhibited both anchorage dependent (IC_50_ of 13-105 nM) and anchorage independent KM-12 cell proliferation (IC_50_ of 45-206 nM). Furthermore, merestinib demonstrated potent anti-tumor effect *in vivo* in multiple xenograft tumor models bearing NTRK gene rearrangements. In both KM-12 xenograft and the PDX model EL1989 harboring a *TPM3-NTRK1* gene fusion, merestinib significantly reduced tumor growth as compared to vehicle or crizotinib treated tumors. In EL1989 PDX, merestinib treatment resulted in tumor regression. Merestinib also significantly reduced tumor growth in a head and neck squamous cell carcinoma (HNSCC) PDX model expressing *ETV6-NTRK3* gene fusion. Together these data suggest that merestinib blocks p-NTRK signaling and blocks tumor growth in oncogenic driven NTRK gene rearranged tumors.

While targeted tyrosine kinase inhibitors such as EGFR and ALK inhibitors, often yield early clinical response, they are frequently not durable due to onset of acquired resistance of secondary mutations in the kinase domain. Similar experiences of acquired resistance to experimental NTRK inhibitor treatment in patients with NTRK fusion have already been reported for entrectinib and larotrectinib [8–10]. The reported secondary acquired mutations are G623R in NTRK3 fusion and G595R and G667C mutations in NTRK1 fusions. We compared the potency of the type II NTRK1 inhibitor, merestinib, with several type I NTRK inhibitors (entrectinib, larotrectrinib/LOXO-101 and crizotinib) on p-NTRK1 signaling in NIH-3T3 cells stably expressing either wild-type, G595R or G667C mutated *TPM3-NTRK1*. All NTRK inhibitors eliminated p-NTRK signaling in wild-type expressing *TPM3-NTRK1 in vitro*. In the G595R and G667C mutant cell lines, larotrectinib and crizotinib did not show inhibitory effect on NTRK1 phosphorylation. With the G667C mutant, entrectinib or merestinib abolished p-NTRK at 0.2 μM and 0.5 μM. In contrast, only moderate p-NTRK1 inhibition was shown by entrectinib or merestinib in cells expressing the G595R mutation at 0.5 μM. *In vivo*, entrectinib or merestinib treatment resulted in tumor regression in tumors with NIH-3T3 cells stably expressing wild-type *TPM3-NTRK1*. Merestinib, but not entrectinib treatment also resulted in sustained tumor regression in tumors with NIH-3T3 cells stably expressing G667C mutant *TPM3-NTRK1*. Neither entrectinib nor merestinib significantly inhibited tumor growth of the mutant G595R *TPM3-NTRK1 in vivo*. These data suggest that complete and not partial p-NTRK inhibition is necessary to block tumor growth, and that merestinib, a type II NTRK kinase inhibitor may have an advantage over type I NTRK kinase inhibitors in durability of treatment response in patients.

Modeling and crystal structure of bound entrectinib and merestinib also provide insight into the difference in the *in vivo* data of the two compounds in this study. The G595R and G667C mutations contributing to entrectinib resistance are much closer in distance to the bound entrectinib or crizotinib than to merestinib. Similar short distance of the bound larotrectinib to G595 and G667 was shown recently [9]. Thus the steric hindrance of the G667 mutation to entrectinib in binding to NTRK1 does not predict the similar potency of entrectinib and merestinib in the G667C *TPM3-NTRK1* expressing cell lines *in vitro* as determined by western blot. The structural modeling appears to be a better predictor of the *in vivo* resistance to entrectinib treatment and that merestinib retains potency to the tumors bearing the G667C mutation. It is not known whether NTRK mediated reduction of phosphorylated ERK could also play a role in countering resistance. Merestinib inhibits p-ERK in a dose dependent manner, which is not observed with either entrectinib or larotrectinib/LOXO-101. It is not clear why crizotinib is reducing p-ERK to a greater extent than p-NTRK in either G595R or G667C mutant expressing NIH-3T3 cells.

It is important to point out that while merestinib is a potent inhibitor of NTRK, it also targets additional kinases such as the TAM receptors (AXL, MERTK, and TYRO3), and MKNK1 and MKNK2, which may also contribute to anti-tumor growth. TAM receptor signaling has been implicated in stimulating cancer growth by augmenting pro-survival pathways and diminishing apoptosis [28]. Importantly, we did not detect AXL protein expression in KM-12 cells (data not shown). Antibodies directed against TYRO3 or MERTK are of poor quality and not reliable to assess merestinib induced inhibition. Because eIF4E phosphorylation resides at a convergent point between two predominant signaling pathways (mTOR and ERK signaling), the MKNK kinases play a critical role in the downstream translation initiation of pro-cancer mRNA [16]. Shown here, merestinib and its metabolites inhibit phosphorylation of eIF4E via MKNK1 and MKNK2 in KM-12 cells. Understanding MKNK inhibition is an ongoing interest and merestinib’s contribution to curbing translation initiation is currently being explored.

Together, these data indicate that merestinib is a potent inhibitor of NTRK and blocks tumor progression *in vivo* in preclinical studies. These data support the clinical evaluation of merestinib in patients with NTRK rearrangements (NCT02920996). Merestinib as a type II NTRK kinase inhibitor may also offer an advantage over type I NTRK kinase inhibitors in retaining potency to acquired secondary kinase mutations, similar to the hypothesis for type I and type II MET kinase inhibitors for MET driven tumors [29, 30]. Merestinib may also offer as an alternative to LOXO-195, a second generation NTRK inhibitor designed to overcome acquired resistance to treatment with type I NTRK inhibitors [9].

## MATERIALS AND METHODS

Kinase activity profiling for merestinib, M1 and M2 metabolites were analyzed using the scanMax Kinase Assay Panel at 0.2, 1 and 5 μM concentrations with % inhibition calculated as described by DiscoveRx (Freemont, CA). Subsequently, the binding affinity (Kd) for merestinib, M1 and M2 metabolites was determined using an 11-point concentration response curve for TrkA, B, C (NTRK1, 2, 3). The TrkA PathHunter cell based kinase assay was performed at DiscoveRx. All *in vivo* experimental protocols were approved by the Eli Lilly and Company Animal Care and Use Committee. Eli Lilly and Company is accredited by the Association for Assessment and Accreditation of Laboratory Animal Care International. Please refer to the Supplementary Materials and Methods for a detailed description of the following: anchorage dependent and independent cell proliferation of KM-12 cells; western blot analysis; PCR and DNA sequence verification of NTRK fusions; merestinib co-crystal structural analysis; *in vivo* mouse xenograft studies; cloning and cell transfection of wild-type *TPM3-NTRK1* and *TPM3-NTRK1* kinase domain mutants; histological assessment of xenograft tumors; imaging and quantification of markers in xenograft tumors.

## SUPPLEMENTARY MATERIALS FIGURES AND TABLES



## References

[1] Parker BC, Zhang W (2013). Fusion genes in solid tumors: an emerging target for cancer diagnosis and treatment. Chin J Cancer.

[2] Malik SM, Maher VE, Bijwaard KE, Becker RL, Zhang L, Tang SW, Song P, Liu Q, Marathe A, Gehrke B, Helms W, Hanner D, Justice R (2014). U.S. Food and drug administration approval: crizotinib for treatment of advanced or metastatic non-small cell lung cancer that is anaplastic lymphoma kinase positive. Clin Cancer Res.

[3] Roskoski R (2017). Anaplastic lymphoma kinase (ALK) inhibitors in the treatment of ALK-driven lung cancers. Pharmacol Res.

[4] Jabbour E (2016). Chronic myeloid leukemia: first-line drug of choice. Am J Hematol.

[5] Huang EJ, Reichardt LF (2003). Trk receptors: roles in neuronal signal transduction. Ann Rev Biochem.

[6] Vaishnavi A, Le AT, Doebele RC (2015). TRKing down an old oncogene in a new era of targeted therapy. Cancer Discov.

[7] Wong V, Pavlick D, Brennan T, Yelensky R, Crawford J, Ross JS, Miller VA, Malicki D, Stephens PJ, Ali SM, Ahn H (2016). Evaluation of a congenital infantile fibrosarcoma by comprehensive genomic profiling reveals an LMNA-NTRK1 gene fusion responsive to crizotinib. J Nat Cancer Institute.

[8] Drilon A, Siena S, Ou SI, Patel M, Ahn MJ, Lee J, Bauer TM, Farago AF, Wheler JJ, Liu SV, Doebele R, Giannetta L, Cerea G (2017). Safety and antitumor activity of the multitargeted pan-TRK, ROS1, and ALK inhibitor entrectinib: combined results from two phase i trials (ALKA-372-001 and STARTRK-1). Cancer Discov.

[9] Drilon A, Nagasubramanian R, Blake JF, Ku N, Tuch BB, Ebata K, Smith S, Lauriault V, Kolakowski GR, Brandhuber BJ, Laresen PD, Bouhana KS, Winski SL (2017). A next-generation TRK kinase inhibitor overcomes acquired resistance to prior TRK kinase inhibition in patients with TRK fusion-positive solid tumors. Cancer Discov.

[10] Russo M, Misale S, Wei G, Siravegna G, Crisafulli G, Lazzari L, Corti G, Rospo G, Novara L, Mussolin B, Bartolini A, Cam N, Patel R (2016). Acquired resistance to the TRK inhibitor entrectinib in colorectal cancer. Cancer Discov.

[11] Sartore-Bianchi A, Ardini E, Bosotti R, Amatu A, Valtorta E, Somaschini A, Raddrizzani L, Palmeri L, Banfi P, Bonazzina E, Misale S, Marrapese G, Leone A (2015). Sensitivity to entrectinib associated with a novel LMNA-NTRK1 gene fusion in metastatic colorectal cancer. J Natl Cancer Inst.

[12] Yan SB, Peek VL, Ajamie R, Buchanan SG, Graff JR, Heidler SA, Hui YH, Huss KL, Konicek BW, Manro JR, Shih C, Stewart JA, Stewart TR (2013). LY2801653 is an orally bioavailable multi-kinase inhibitor with potent activity against MET, MST1R, and other oncoproteins, and displays anti-tumor activities in mouse xenograft models. Invest New Drugs.

[13] Morikawa K, Walker SM, Jessup JM, Fidler IJ (1988). *In Vivo* selection of highly metastatic cells from surgical specimens of different primary human colon carcinomas implanted into nude mice. Cancer Res.

[14] Ardini E, Bosotti R, Borgia AL, De Ponti C, Somaschini A, Cammarota R, Amboldi N, Raddrizzani L, Milani A, Magnaghi P, Ballinari D, Casero D, Gasparri F (2014). The TPM3-NTRK1 rearrangement is a recurring event in colorectalcarcinoma and is associated with tumor sensitivity to TRKA kinase inhibition. Mol Oncol.

[15] Taipale M, Krykbaeva I, Whitesell L, Santagata S, Zhang J, Liu Q, Gray NS, Lindquist S (2013). Chaperones as thermodynamic sensors of drug-target interactions reveal kinase inhibitor specificities in living cells. Nat Biotechnol.

[16] Siddiqui N, Sonenberg N (2015). Signaling to eIF4E in cancer. Biochem Soc Trans.

[17] Ardini E, Menichincheri M, Banfi P, Bosotti R, De Ponti C, Pulci R, Ballinari D, Ciomei M, Texido G, Degrassi A, Avanzi N, Amboldi N, Saccardo MB (2016). Entrectinib, a pan-TRK, ROS1, and ALK inhibitor with activity in multiple molecularly defined cancer indications. Mol Cancer Ther.

[18] Roskoski R (2016). Classification of small molecule protein kinase inhibitors based upon the structures of their drug-enzyme complexes. Pharmacol Res.

[19] Roskoski R (2017). ROS1 protein-tyrosine kinase inhibitors in the treatment of ROS1 fusion protein-driven non-small cell lung cancers. Pharmacol Res.

[20] Huang Q, Johnson TW, Bailey S, Brooun A, Bunker KD, Burke BJ, Collins MR, Cook AS, Cui JJ, Dack KN, Deal JG, Deng YL, Dinh D (2014). Design of potent and selective inhibitors to overcome clinical anaplastic lymphoma kinase mutations resistant to crizotinib. J Med Chem.

[21] Pulciana S, Santos E, Lauver AV, Aaronson A, Barbacid M (1982). Oncogenes in solid human tumours. Nature.

[22] Martin-Zanca D, Mitra G, Long LK, Barbacid M (1986). Molecular characterization of the human trk oncogene. Cold Spring Harb Symp Quant Biol.

[23] Hirose M, Kuroda Y, Murata E (2016). NGF/TrkA signaling as a therapeutic target for pain. Pain Pract.

[24] Khan N, Smith MT (2015). Neurotrophins and neuropathic pain: role in pathobiology. Molecules.

[25] Rosenthal A, Lin JC (2014). Modulation of neurotrophin signaling by monoclonal antibodies. Handb Exp Pharmacol.

[26] Bradshaw RA, Chalkley RJ, Biarc J, Burlingame AL (2013). Receptor tyrosine kinase signaling mechanisms: devolving TrkA responses with phosphoproteomics. Adv Biol Regul.

[27] Mitchell DJ, Blasier KR, Jeffery ED, Ross MW, Pullikuth AK, Suo D, Park J, Smiley WR, Lo KW, Shabanowitz J, Deppmann CD, Trinidad JC, Hunt DF (2012). Trk activation of the ERK1/2 kinase pathway stimulates intermediate chain phosphorylation and recruits cytoplasmic dynein to signaling endosomes for retrograde axonal transport. J Neurosci.

[28] Verma A, Warner SL, Vankayalapati H, Bearss DJ, Sharma S (2011). Targeting Axl and Mer kinases in cancer. Mol Cancer Ther.

[29] Bahcall M, Sim T, Paweletz CP, Patel JD, Alden RS, Kuang Y, Sacher AG, Kim ND, Lydon CA, Awad MM, Jaklitsch MT, Sholl LM, Janne PA (2016). Acquired MET^D1228V^ mutation and resistance to MET inhibition in lung cancer. Cancer Discov.

[30] Ou SI, Young L, Schrock AB, Johnson A, Klempner SJ, Zhu VW, Miller VA, Ali SM (2017). Emergence of pre-existing MET Y1230C mutation as a resistance mechanism to crizotinib in NSCLC with MET exon 14 skipping. J Thorac Oncol.

