# Supplementary material for: Merestinib (LY2801653) inhibits neurotrophic receptor kinase (NTRK) and suppresses growth of NTRK fusion bearing tumors
Source: Oncotarget. 2018 Feb 13;9(17):13796–806. doi: 10.18632/oncotarget.24488 (PMC5862616; doi:10.18632/oncotarget.24488)
Supplement: Supplementary file 1 [file oncotarget-09-13796-s001.pdf]

Merestinib (LY2801653) inhibits neurotrophic receptor kinase (NTRK) and suppresses growth of NTRK fusion bearing tumors

SUPPLEMENTARY MATERIALS

Kinase profiling

Merestinib, M1 and M2 metabolites were analyzed using the scanMax Kinase Assay Panel at 0.2, 1 and 5 µM concentrations with % inhibition calculated as described by DiscoveRx (Freemont, CA). Subsequently, the binding affinity (Kd) for merestinib, M1 and M2 metabolites was determined using a 10-point concentration response curve for TrkA, B, C (NTRK1, 2, 3). The TrkA PathHunter cell based kinase assay was performed at DiscoveRx.

Anchorage dependent and independent cell proliferation

KM-12 cells were acquired from the NCI-Frederick Cancer DCTC Repository and cultured in RPMI media supplemented with 10% fetal bovine serum (FBS) and were plated in 96 well plates at 4000 cells/well. The following day, culture media was replaced with 2% FBS containing media. Cells were treated with merestinib, M1, or M2 metabolite at a dose concentration from 0.0003 µM to 10 µM for 72 hours. CellTiter-Glo® was added according to manufacturer's protocol (Promega, Madison WI). Cell viability was determined by measuring relative luminescence unit (RLU) using a Wallac Victor^2 1420 multilable counter (Perkin Elmer, Waltham MA). DMSO treated cells served as the reference comparator (0% inhibition) while 10 µM staurosporin was used for normalization defined as 0% control. Values recorded as % control were subsequently plotted using GraphPad Prism 7 with IC_{50} calculations based on 4-parameter curve-fit analysis.

Assessment of anchorage independent growth of KM-12 cells treated with merestinib, M1, M2 and crizotinib was performed using the S+ Chip Analyzer (Samsung Electro-Mechanics Co. Ltd., South Korea) as described in detail previously [1]. Briefly, 80 cells were spotted in the presence of RPMI-1640 media containing 2% FBS (final) onto microwells with alginate hydrogel added to a final concentration of 0.5% with inhibitor concentration ranging from 0.17 nM to 10,000 nM. DMSO served as the untreated control. Cells were incubated with compounds for 3 days, calcein stained and subsequently scanned. IC_{50} calculations using variable slope (4-parameter) of log inhibitor concentration versus response were performed in GraphPad Prism.

In vivo mouse studies

For KM-12 cell-derived xenograft studies, 5 million cells were implanted in the rear flank of athymic nu/nu mice (Envigo, Indianapolis, IN). Dosing began once average tumor volume reached 200 mm^3. The colorectal PDX model EL1989 was originally established from a pT3a tumor of the colon excised from an 80 year old male Caucasian patient. Presence of the *TPM3-NTRK1* genomic rearrangement in this tumor was identified through exome and RNA sequencing analysis. This was independently confirmed through RT-PCR analysis of the tumor. EL1989 tumor was propagated in athymic nude mice, harvested, sectioned into small fragments and subsequently implanted in nude mice. Merestinib and crizotinib treatment began once average tumor volume reached 150 mm^3. Merestinib was formulated in 10% PEG 400 (Fisher Chemical, Pittsburgh PA)/90% (20% Captisol) (Cydex Pharmaceuticals, San Diego CA), administered orally at 24 mg/kg once daily. Crizotinib formulated in 10% Acacia and 0.05% antifoam (Dow Chemical, Bristol PA) was administered orally at 25 mg/kg twice daily. Entrectinib (MedKoo Biosciences, Inc., Morrisville NC) was formulated in 0.5% methylcellulose, 1% Tween 80 in water and administered orally at 30 mg/kg twice daily. Animal body weights were recorded twice weekly. All *in vivo* experimental protocols were approved by the Eli Lilly and Company Animal Care and Use Committee. Eli Lilly and Company is accredited by the Association for Assessment and Accreditation of Laboratory Animal Care International. The *in vivo* efficacy study involving the CTG-0798 HNSCC PDX model harboring an *ETV6-NTRK3* fusion (Supplementary Figure 7) was performed at Champions Oncology (Hackensack, NJ). CTG-0798 tumors were propagated in immunocompromised nude mice and tumor fragments were implanted in athymic nude mice for the study. Compound treatment began once average tumor volume reached 150-300 mm^3. Statistical analysis was performed as described previously [2].

Cloning and cell transfection

Wild-type *TPM3-NTRK1* as well as the NTRK1 kinase domain G595R and G667C mutants were cloned, including a 3' 3X FLAG-Tag inserted into a pcDNA3.1 vector backbone. NIH-3T3 cells were transfected using Lipofectamine™ LTX (ThermoFisher Scientific, Grand Island, NY). Stable clonal cell pools were generated after

350 μg/ml hygromycin selection for 14 days. Each stable pool was DNA sequenced confirming the appropriate wild-type or mutated NTRK1. Control vector containing eGFP served as a negative control.

Western blotting

After treatment, KM-12 cells were washed once with dPBS and harvested with 200 μL cell extraction buffer (ThermoScientific, CA) in the presence of HALT protease and phosphatase inhibitors (Pierce, Rockford, IL). 20 μg cell lysates were electrophoretically separated, transferred to PVDF membrane, and blocked in StartingBlock T20 (ThermoScientific, Rockford, IL) for 30 minutes. Membranes were treated with the following antibodies diluted in 5% BSA in 1X TBST: p-NTRK Y490 (1:500) (Sigma-Aldrich, St. Louis, MO), total NTRK (1:500) (Abcam, Cambridge, MA), p-ERK (1:1000) or total ERK (1:1000) (Cell Signaling Technology, Danvers, MA), p-eIF4E S209 (1:1000) (Abcam, Cambridge, MA), total eIF4E (1:1000) (Becton-Dickenson, Franklin Lakes, NJ), β-Actin (1:10,000) (Sigma-Aldrich), GAPDH (1:2000) (Cell Signaling Technology, Danvers, MA), FLAG-M2 (1:2000) (Sigma-Aldrich), HRP-conjugated mouse or rabbit secondary antibodies (1:2000) (GE Healthcare, Little Chalfont, UK). All western blots were developed using West-Pico chemiluminescent substrate (ThermoScientific) and images captured on a Roche-Lumi-Imager.

Histological assessment of PDX tumors

Subcutaneous tumors were collected in 10% neutral buffered formalin, fixed, processed and embedded in paraffin. Tumor samples were trimmed to provide the maximum tissue area in the histologic section. Sections were made at 4 microns and were stained with hematoxylin and eosin. Slides were evaluated qualitatively by a board-certified pathologist (KMC).

Immunofluorescence and iCys imaging and quantitation

EL1989 PDX tumors were processed to paraffin blocks and sections were made as described above. Slides were baked at 60°F for 1 hour and then deparaffinized in xylene (4 × 10 minutes); rehydrated with ethanol/water immersions with final washes in TBST; blocked with Protein Block (Dako, Santa Clara CA) for 30 minutes; stained with a combination of Hoechst 33324 and Ki67 (Thermo Scientific RM9106, clone SP6)/anti-rabbit Alexa Fluor-647 (Invitrogen, Carlsbad CA) then imaged using an iCys Laser Scanning Cytometer (CompuCyte, Newton NJ) and a Marianas Digital Imaging Workstation configured with a Zeiss Axiovert 200M inverted fluorescence microscope (Intelligent Imaging Innovations, Denver CO). Percent Ki67+ area was calculated as the percentage of total tissue area (Hoechst positive) that is also Ki67+ positive. Quantitative data comparisons of treatment groups were done using the Dunnett's analysis in JMP statistics software (SAS).

PCR and DNA sequence verification of NTRK fusions

Material and method for EL1989 and KM-12 *TPM3-NTRK1* fusion confirmation

RNA was extracted from frozen EL1989 tumor section and KM-12 cell line using Direct-zol™ RNA MiniPrep cat# R2051 following manufacturer's protocol. cDNA was prepared from 1 ug RNA in 20 uL volume using SuperScript VILO MasterMix (Invitrogen, Carlsbad CA). mRNA sequences of TPM3 (NM_153649.3) and NTRK1 (NM_001012331.1) were obtained from NCBI for primer design. A forward primer upstream of the *TPM3* fusion junction (as previously identified from RNAseq data) and a reverse primer downstream of the *NTRK1* fusion junction were designed with online program Primer3 for PCR and Sanger sequencing primers. The primer sequences are TPM3_Ex3B_F5: AGGCAGATAGGAAGTATGAAGAGG, NTRK1_Ex12_R5: AGGCATCACTGAAGTATTGTGG. PCR was carried out with Advantage®2 Polymerase Mix (Clontech, Mountainview CA) with Advantage 2 PCR buffer and cycled at 95°C for 2 minutes; 35 cycles of 95°C for 30 seconds; 65°C for 30 seconds, 72°C for 30 seconds, and a final extension of 72°C for 10 minutes. PCR was also performed for *GAPDH* house-keeping gene as internal control (GAPDH Ex3F: ATCCCATCACCATCTTCCAG, GAPDH Ex7R: CCATCACGCCACAGTTTCC). PCR products were purified with Wizard® SV Gel and PCR Clean-Up System (Promega, Madison WI). Sequencing PCR was carried out using ABI BigDye Terminator v3.1 cycle sequencing kit (Life Technologies, Carlsbad CA). The resulting products were run on an ABI 3730xl DNA analyzer. All sequences were visually analyzed with Sequencher (Gene Codes Corp., Ann Arbor MI) and sequencing results confirmed *TPM3* exon 7 (NM_153649.3) fusion to *NTRK1* exon 9 (NM_001012331.1) in EL1989 PDX model and KM-12 cell line.

Material and method for CTG-0798 *ETV6-NTRK3* fusion confirmation

CTG-0798 tumor frozen section was received from Champions. RNA was extracted using Direct-zol™ RNA MiniPrep following manufacturer's protocol (Zymo Research, Irvine CA). cDNA was prepared from 1 μg RNA in 20 μl volume using SuperScript VILO MasterMix (Invitrogen). mRNA sequences of *ETV6* (NM_001987) and NTRK3 (NM_002530) were obtained

from NCBI for primer design. A forward primer upstream of the *ETV6* fusion junction (as previously identified from RNAseq data) and a reverse primer downstream of the *NTRK3* fusion junction were designed with online program Primer3 for PCR and Sanger sequencing primers. The primer sequences are ETV6_Ex4_F5: CCTGGAAACTCTATACACACACAGC, NTRK3_Ex16D_R5: GGAACTTATTCAGGTCTCCATGC. PCR was carried out with Advantage®2 Polymerase Mix (Clontech) with Advantage 2 PCR buffer and cycled at 95°C for 2 minutes; 35 cycles of 95°C for 30 seconds; 65°C for 30 seconds, 72°C for 30 seconds, and a final extension of 72°C for 10 minutes. PCR was also performed for *GAPDH* house-keeping gene as internal control (GAPDH Ex3F: ATCCCATCACCATCTTCCAG, GAPDH Ex7R: CCATCACGCCACAGTTTCC). PCR products were purified with Wizard® SV Gel and PCR Clean-Up System (Promega #A9281). Sequencing PCR was carried out using ABI BigDye Terminator v3.1 cycle sequencing kit (Life Technologies). The resulting products were run on an ABI 3730xl DNA analyzer. All sequences were visually analyzed with Sequencher (Gene Codes Corp.) and sequencing results confirmed *ETV6* exon 5 (NM_001987) fusion to *NTRK3* exon 15 (NM_002530) in CTG-0798 PDX model.

Co-crystallization of merestinib with NTRK1 kinase and x-ray diffraction analysis

The kinase domain of TrkA containing residues 500-787 was cloned, expressed, and purified as previously described [3]. Purified protein was treated with 1.5 mM compound and crystallized in the space group P64 (a = b = 75.36 Å, c = 113.1 Å) at room temperature by hanging drop vapor diffusion against 2.2 M Sodium Chloride and 100 mM Bis-Tris Propane pH 7.0. Crystals were harvested with 25% glycerol as the cryoprotectant and flash frozen in liquid nitrogen. Diffraction data were collected at the LRL-CAT 31-ID beamline of the Advanced Photon Source, Argonne. The structure was solved by molecular replacement using the program PHASER [4] and Protein Data Bank entry 4PMM.pdb as a starting model [3]. The structure was subject to iterative rounds of manual rebuilding with the program COOT [5] and restrained refinement using the progam autoBUSTER (Global Phasing Ltd, Cambridge, U.K.). The final structure has crystallographic R/Rfree values of 0.173/0.207 at a resolution of 1.94 Å, and merestinib binding is unambiguously defined by the electron density maps.

ACKNOWLEDGMENTS

This research used resources of the Advanced Photon Source, a U.S. Department of Energy (DOE) Office of Science User Facility operated for the DOE Office of Science by Argonne National Laboratory under Contract No. DE-AC02-06CH11357.

Use of the Lilly Research Laboratories Collaborative Access Team (LRL-CAT) beamline at Sector 31 of the Advanced Photon Source was provided by Eli Lilly Company, which operates the facility.

REFERENCES

1. Lee DW, Choi YS, Seo YJ, Lee MY, Jeon SY, Ku B, Kim S, Yi SH, Nam DH. High-throughput screening (HTS) of anticancer drug efficacy on a micropillar/microwell chip platform. Anal Chem. 2014; 86:535–542.
2. Yan SB, Peek VL, Ajamie R, Buchanan SG, Graff JR, Heidler SA, Hui YH, Huss KL, Konicek BW, Manro JR, Shih C, Stewart JA, Stewart TR, et al. LY2801653 is an orally bioavailable multi-kinase inhibitor with potent activity against MET, MST1R, and other oncoproteins, and displays anti-tumor activities in mouse xenograft models. Invest New Drugs. 2013; 31:833–844.
3. Stachel SJ, Sanders JM, Henze DA, Rudd MT, Su HP, Li Y, Nanda NN, Egbertson MS, Manley PJ, Jones KL, Brnardic EJ, Green A, Grobler JA, et al. Maximizing diversity from a kinase screen: identification of novel and selective pan-Trk inhibitors for chronic pain. J Med Chem. 2014; 57:5800–5816.
4. McCoy AJ, Grosse-Kunstleve RW, Adams PD, Winn MD, Storoni LC, Read RJ. Phaser crystallographic software. J Appl Cryst. 2007; 40:658–674.
5. Emsley P, Lohkamp B, Scott WG, Cowtan K. Features and development of coot. Acta Crystallographica Section D. 2010; 66:486–501.

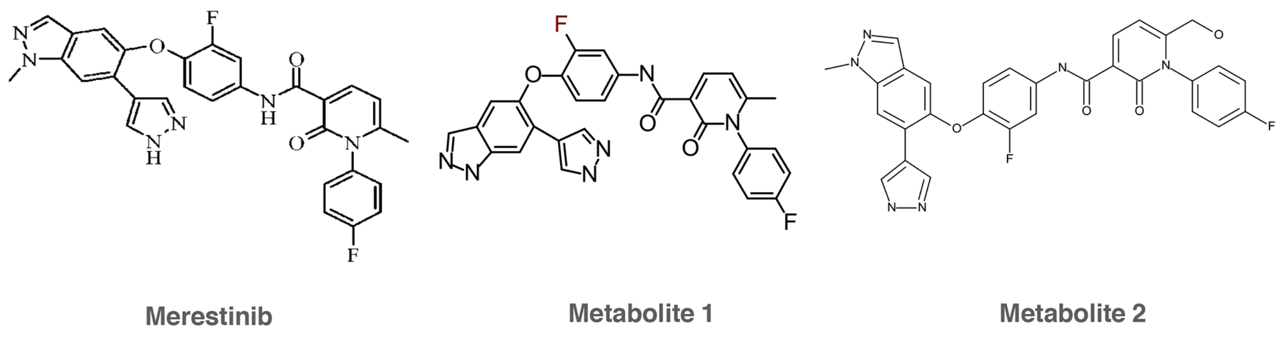

Supplementary Figure 1: Chemical structure of merestinib and its two metabolites.

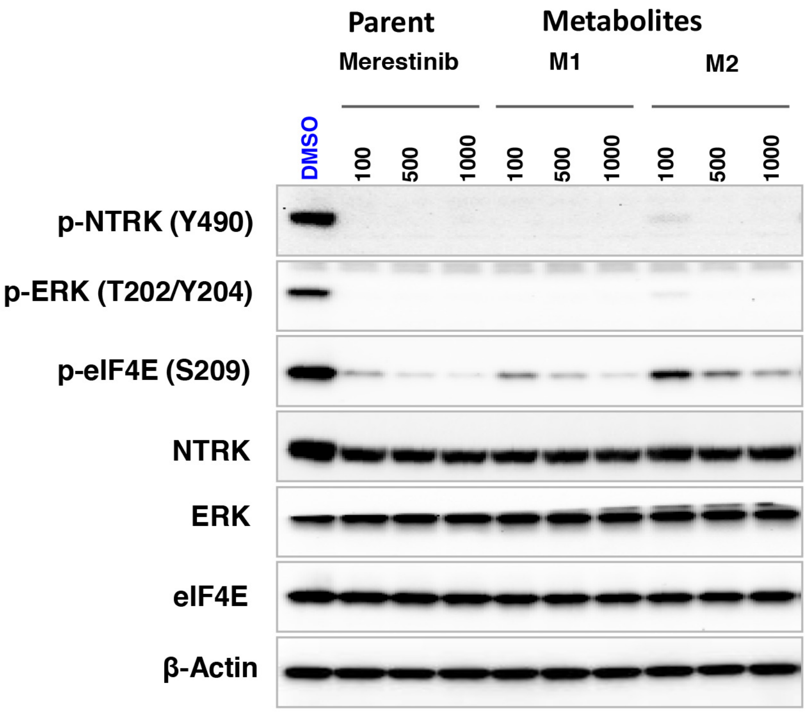

Supplementary Figure 2: Western blot analysis of KM-12 cells treated with the metabolites of merestinib. KM-12 cells treated for 2 hours with merestinib, or its metabolites M1 or M2 at 100, 500, 1000 nM.

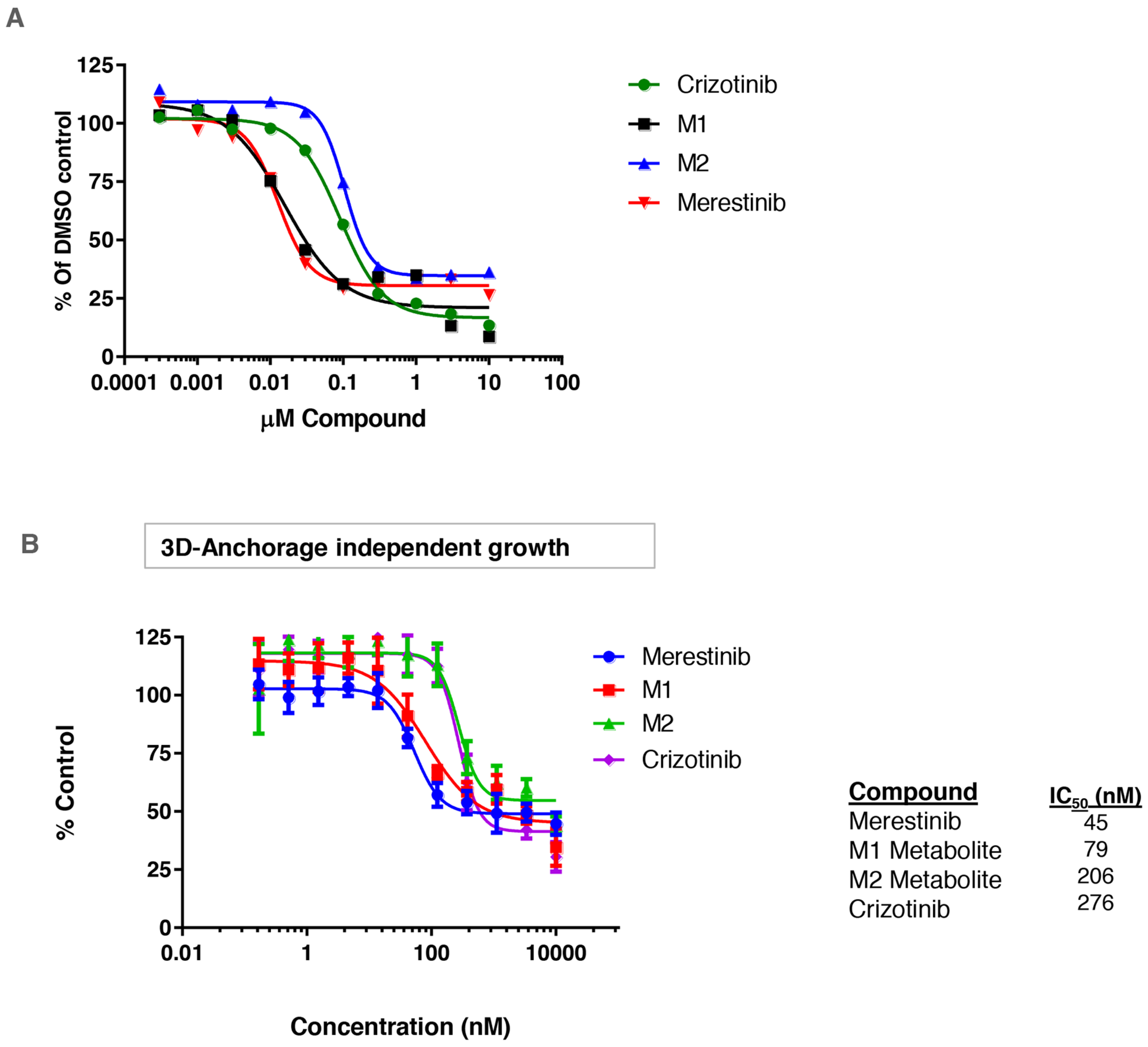

Supplementary Figure 3: *In vitro* effect of merestinib and its metabolites on the proliferation of KM-12 cells. (A) Proliferating KM-12 cells were treated with merestinib, metabolites or crizotinib for 96 hours. Cell viability was determined by CellTiter-Glo® as described. Percent control was calculated based on DMSO control. IC_{50} calculations performed in GraphPad Prism. Data in figure were representative from 3 independent experiments. **(B)** Representative graph of 3-Dimension growth of KM-12 cells cultured in alginate hydrogel and treated with merestinib, M1, M2 or crizotinib (0.17 nM – 10 µM) for 3 days and quantified.

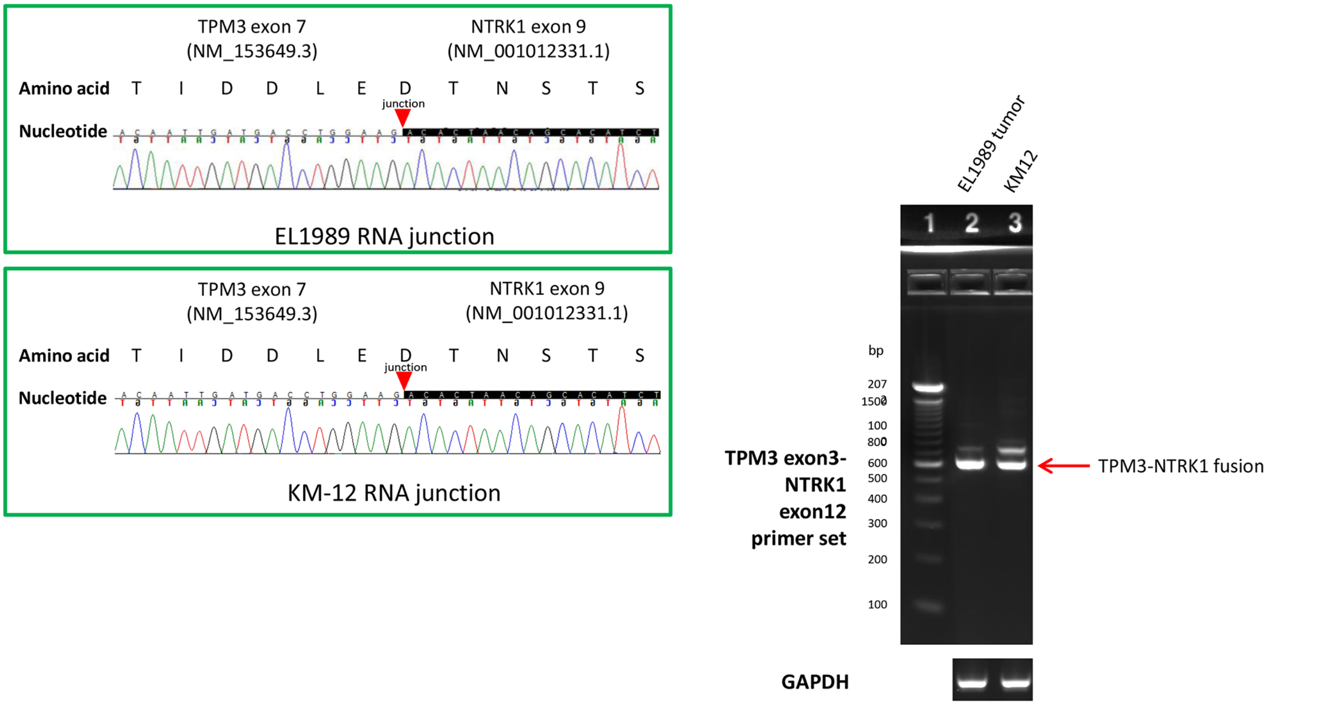

Supplementary Figure 4: PDX model EL1989 containing *TPM3-NTRK1* fusion determined by RT-PCR. *TPM3-NTRK1* fusion in EL1989 sequence aligns identically with the KM-12 cell line containing the *TPM3-NTRK1* fusion.

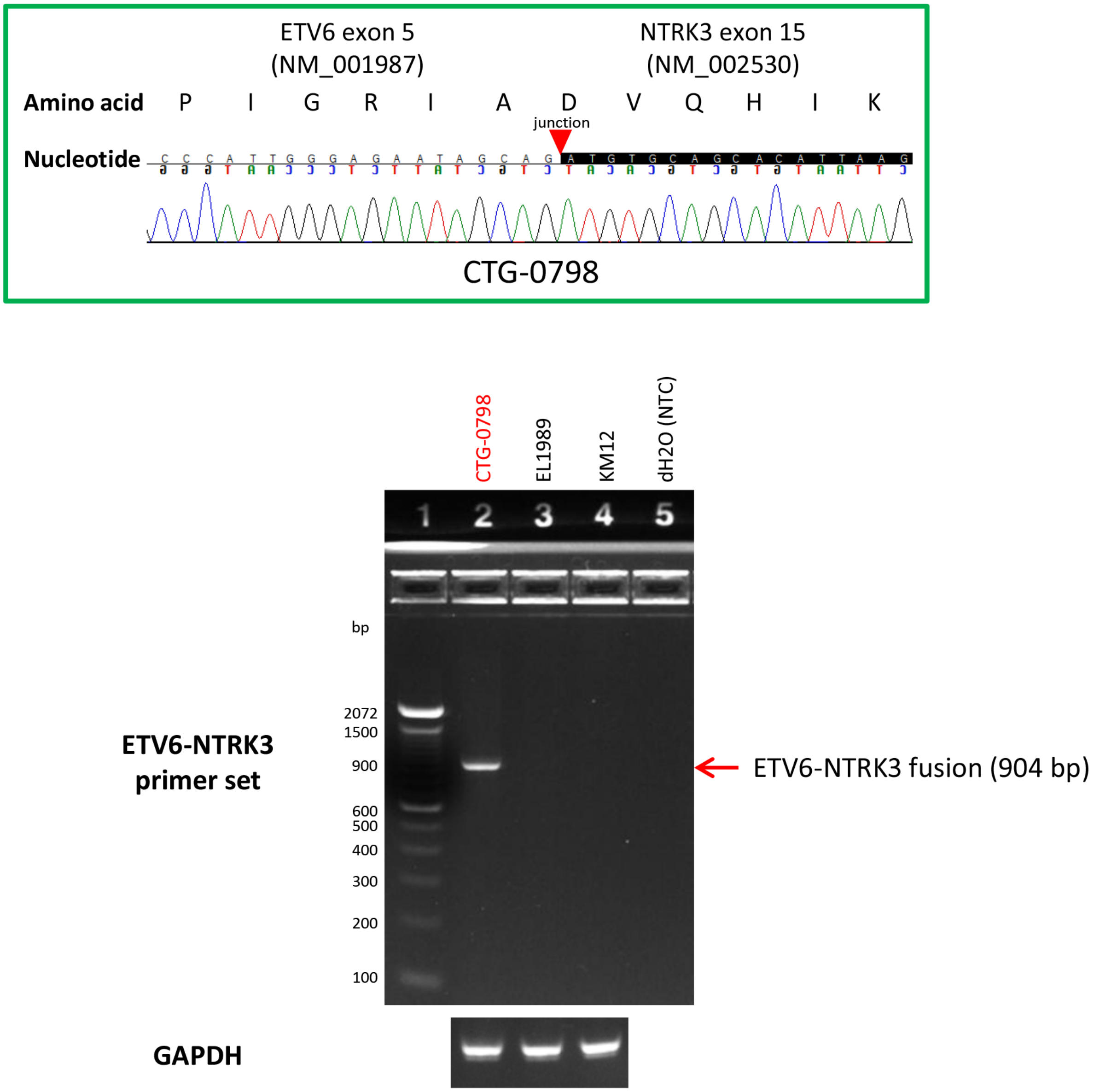

Supplementary Figure 5: DNA Sanger sequencing confirmation of the *ETV6-NTRK3* gene fusion present in the HNSCC PDX model CTG-0798.

Construct	NTRK1 (G595)	NTRK1 (G667)
Wild-Type	R H **G** D L<br>CGG CAC **GGG** GAC CTC	V K I **G** D<br>GTC AAG ATT **GGT** GAT
G595	R H **R** D L<br>CGG CAC **AGG** GAC CTC	V K I **G** D<br>GTC AAG ATT **GGT** GAT
G667	R H **G** D L<br>CGG CAC **GGG** GAC CTC	V K I **C** D<br>GTC AAG ATT **TGT** GAT

Supplementary Figure 6: Sequencing to confirm constructs of wild-type, G667C or G595R mutant variants in *TPM3-NTRK1*. DNA sequencing confirming *TPM3-NTRK1* constructs of wild-type, G667C or G595R mutant variants in stably transfected NIH-3T3 cells. RNA isolated from each stable pool was sequenced in both forward and reverse primers as described in the Supplementary Materials and Methods section.

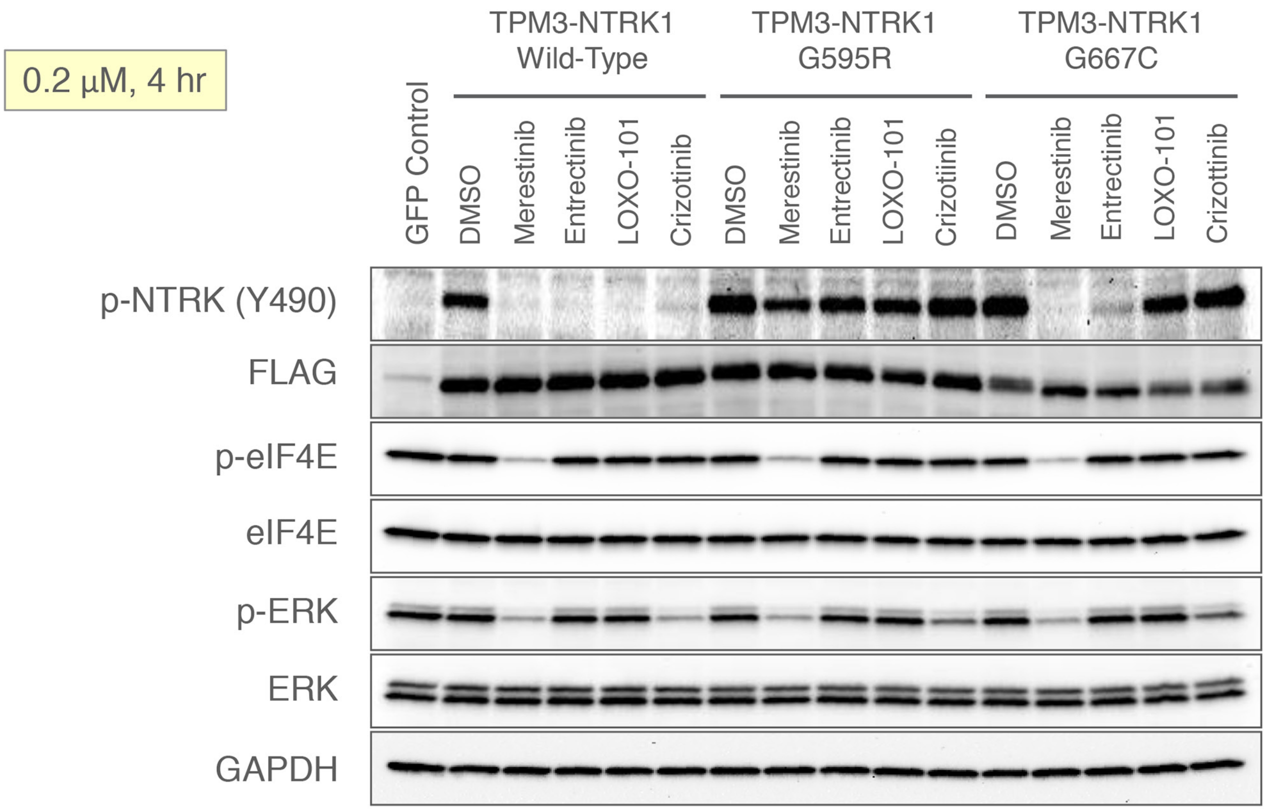

Supplementary Figure 7: Evaluation of NTRK inhibitors with NIH-3T3 cells transfected with G595R or G667C mutation in *TPM3-NTRK1* fusion *in vitro*. Cell lysates from NIH-3T3 cells stably transfected with *TPM3-NTRK1* wild-type, mutant G595R, or G667C *TPM3-NTRK1* expressing clones were analyzed by immunoblotting after treatment with 0.2 µM of the indicated NTRK inhibitor for 4 hours. All three *TPM3-NTRK1* clones expressed 3′-3X-FLAG-Tag as confirmed by anti-FLAG antibody. eGFP control vector served as a control with no NTRK or FLAG expression.

Supplementary Table 1: Merestinib and two primary metabolites exhibit similar kinase activity profiles

	ScanMax™ kinase panel assay % Inhibition at 0.2 µM		
**Kinase**	**Merestinib**	**M1**	**M2**
**MET**	98	100	93
**MST1R**	96	95	88
**AXL**	100	99	100
**ROS1**	83	95	78
**MKNK1**	56	70	62
**MKNK2**	98	98	95
**PDGFRA**	55	80	70
**FLT3**	95	96	97
**MERTK**	96	99	93
**TYRO3**	99	100	97
**TEK (TIE2)**	99	100	100
**DDR1**	100	100	100
**DDR2**	97	94	99
**CSF1R**	97	98	97
**VEGFR2**	63	72	43
**RET**	79	72	61

Supplementary Table 2: Estimated proportion of viable tumor cells, necrotic material and mucin content according to each histological section per group. Colorectal carcinoma PDX EL1989 tumors were collected at the end of study on Day 80, 28 days of treatment.

Group-Animal ID	% Viable tumor	% Necrosis/Mucin
Vehicle-1	60%	40%
Vehicle-2	80%	20%
Vehicle-3	5%	95%
Vehicle-4	60%	40%
Vehicle-5	30%	70%
Vehicle-6	80%	20%
Vehicle-7	10%	90%
Vehicle-8	80%	20%
Vehicle-9	90%	10%
Vehicle-10	10%	90%
Mean	50%	50%
Merestinib-1	5%	95%
Merestinib-2	30%	70%
Merestinib-3	10%	90%
Merestinib-4	10%	90%
Merestinib-5	70%	30%
Merestinib-6	20%	80%
Mean	25%	75%
Crizotinib-1	70%	30%
Crizotinib-2	90%	10%
Crizotinib-3	20%	80%
Crizotinib-4	80%	20%
Crizotinib-5	5%	95%
Crizotinib-6	20%	80%
Mean	50%	50%
